# Twelve-Month Follow-Up of Different Dentinal Hypersensitivity Treatments by Photobiomodulation Therapy, Nd:YAG and Nd:YAP Lasers

**DOI:** 10.3390/life12121996

**Published:** 2022-11-30

**Authors:** Samir Nammour, Marwan El Mobadder, Melanie Namour, Aldo Brugnera Junior, Fatima Zanin, Ana Paula Brugnera, Sabine Geerts, Amaury Namour

**Affiliations:** 1 Department of Dental Science, Faculty of Medicine, University of Liege, 4000 Liege, Belgium; 2 Dental Surgery Department, Wroclaw Medical University, 50-425 Wroclaw, Poland; 3 Biophotonics Center, Institute Brugnera e Zanin, Sao Paolo 01434-000, Brazil

**Keywords:** laser, dentinal hypersensitivity, restorative dentistry, sensitivity, dental pain, Nd:YAG, Nd:YAP, photobiomodulation

## Abstract

In this retrospective study, data for three different laser-assisted approaches for the management of dentinal hypersensitivity (DH) was collected (*n* = 920 teeth). In total, 387 teeth were treated with photobiomodulation (PBM) therapy with 660 nm red light laser (PBM group), 327 were treated with the Nd:YAG laser (1064 nm) and 206 were treated with the Nd:YAP laser (1340 nm). To assess the effectiveness of each treatment, a visual analogue scale (VAS) was used, where zero represented no pain at all and ten represented the greatest pain. VAS was used before (T0), immediately after (T1), one week after (T2), four weeks after (T3), six months after (T4) and one year after treatment (T5). Means and standard deviations of VAS at different follow-up times were calculated. Values were compared within and between groups. Statistical significance was considered to be achieved when *p*-value was less than 0.05. Confidence level was proposed to be 99% with a P value lower than 0.001. Within groups, a statistically significant reduction was obtained when the mean value of VAS at T0 was compared with T5. At T5, the PBM group had the highest reduction of VAS (with mean value of 0), while the Nd:YAG and Nd:YAP groups had scores of 1.065 ± 0.674 and 4.665 ± 0.674, respectively. Conclusion: this retrospective study showed that PBM therapy and irradiation with Nd:YAG and Nd:YAP lasers are effective in managing DH pain. However, PBM therapy was the only procedure that showed complete pain relief at six and twelve months after treatment.

## 1. Introduction

Dentinal hypersensitivity (DH) is a common oral condition characterized by short, reversible, and acute sensation of pain that is provoked by a stimulus on exposed or naked dentin [1,2]. To date, Brännström’s hydrodynamic theory is commonly accepted to explain the mechanism of action leading to dentinal hypersensitivity [3]. Brännström stated in 1963 that a stimulus on an exposed dentin will result in fluid movements inside dentinal tubules, which will stimulate the nerves endings, leading to the sensation of pain [3]. In fact, the intratubular myelinated A-β, and some A-δ fibers, respond to stimulus from exogenic factors with a short, sharp and reversible pain which characterizes the sensation of pain in DH [3,4,5]. The global average of adults with DH was suggested to be 33.5%, with a prevalence ranging from 1.3% to 92.1%. As for the etiology, studies suggest that DH is multifactorial, with causes involving an association of factors such as friction (abrasion), corrosion (degradation caused by acid from intrinsic and extrinsic sources) and tension (parafunctional habits, traumatic occlusion and malocclusion) [6].

Because dentinal hypersensitivity arises from external factors that stimulate the nerves endings, treatment modalities are focused on carefully but effectively protecting or obliterating dentinal tubules [7,8,9,10]. However, the long-term effectiveness of desensitizing agents used for dentinal hypersensitivity remains a challenge [11].

In this context, a number of desensitizing agents were developed and suggested to be effective options for the management of DH. Among them, potassium ions [12], arginine, calcium carbonate [13], stannous fluoride with and without sodium hexametaphosphate [14,15], oxalates [16] and others are showing promising but heterogenous results. In this context, a network meta-analysis [16] concluded that strontium and potassium result in a moderate effectiveness for tactile stimulus and arginine for air stimulus. There is moderate evidence that the combination of potassium with SnF_2_ or hydroxyapatite can be effective for tactile and air stimuli. Among all active ingredients, calcium sodium phosphosilicate (CSP) was more effective for all three stimuli, with high to moderate certainty. In addition, SnF_2_-based formulations seem to be more effective than regular fluoride toothpastes in the management of DH, due to tactile and air stimuli with high to moderate certainty [16].

DH can be managed by dental professionals at clinics [17,18,19]. This includes professional application of resin-based materials [18], uses of varnished fluoride and lasers with different protocols and parameters [17,18,19]. In this context, a 980 nm diode laser and 1064 nm Nd:YAG laser within a specific protocol, consisting of applying a graphite paste, were both proven to be effective in the management of dentinal hypersensitivity [10,20]. Irradiation with lasers causes different tissue reaction based on the active medium of the laser, its wavelength and the parameters with which the laser was used [21]. For instance, high-power lasers can produce a melting effect on the surface of the irradiated dentin, obliterating the entrance of dentinal tubules, thus stopping intratubular fluid movement [10,22], while lasers used at low power during photobiomodulation therapy (PBM) act as a biomodulator of cellular responses and can, if effective, promote the reduction of pain levels through a depolarization of nerve fibers and increase in the formation of tertiary dentin [23,24]. The literature shows a lack of studies comparing the effectiveness and long-term stability of laser-assisted protocols for the management of DH.

In view of the above discussion, the aim of this study is to compare the effectiveness of photobiomodulation therapy with 660 nm laser, Nd:YAG laser (1064 nm) and Nd:YAP (1340 nm) laser used for the management of DH with 12 months of follow-up. The null hypothesis is that there is no significant difference in mean values when different laser-assisted protocols were compared at different times of follow-up.

## 2. Materials and Methods

### 2.1. Study Design

A multicenter, one-year, retrospective data collection and analysis was performed. The study population consisted of 920 (*n* = 920) treated teeth of 811 patients with a clinical diagnosis of dentinal hypersensitivity and seeking treatment. The lowest number of patients, 206, was in the third group. The number of patients follows the minimal criteria of achieving a power of 90% and an effect size of 0.3. Standard parameters, including significance level of 0.05, *d*-value *d* = 0.3, 95% confidence interval and 90% power of the study were used to calculate a sample size equal to 191 for each group using G × Power software 3.1 (Kiel University, Kiel, Germany). Moreover, demographic data of the population are found in Table 1. A written informed consent was obtained from all the patients prior to their participation. The decision for DH treatment was made after informing the patient about the steps of the treatment and the possibility of any potential failure and/or complication.

Collection of data was made for three different treatment modalities: (1) photobiomodulation (PBM) therapy performed with red light (660 nm), (2) irradiation with Nd:YAG laser (1064 nm) and (3) irradiation with Nd:YAP laser (1340 nm). This retrospective data collection does not include any new clinical protocol or intervention, consequently, it did not legally require a prior approval from the ethical committee of the University of Liege. Our study respected the guideline and checklist of items, extended from the STROBE statement, that should be reported in observational studies using routinely collected health data (Benchimol et al.) [25]. Additionally, it should be mentioned that in our retrospective data collection, there was no existing data on a group of patients with severe, untreated dentin hypersensitivity for one year postoperatively. Indeed, there is a moral obligation to treat all patients suffering from severe pain due to dentin hypersensitivity. Thus, the comparison was made before any treatment (control groups at baseline inclusion) and at different follow-up times for each of the laser-assisted procedures for the treatment of dentinal hypersensitivity (test groups). Moreover, this retrospective study only included patients who had received one similar treatment protocol with the same wavelength and parameters. In other words, patients who received two treatments with two different wavelengths for two different teeth were excluded.

### 2.2. Inclusion and Exclusion Criteria

#### 2.2.1. Inclusion Criteria

Patients diagnosed with at least one permanent tooth with a cervical exposed dentin, seeking treatment from the related pain and with a severity of pain greater than or equal to five, according to the visual analogue scale (VAS), were deemed eligible for inclusion. For cases of multiple teeth with DH for the same patient, data was included only for patients that underwent the same protocol with the same wavelength, in order to avoid any bias.

#### 2.2.2. Exclusion Criteria

Patients with teeth showing evidence of irreversible pulpitis or necrosis, carious lesions, defective restorations, facets of attrition, premature contact, cracked enamel and active periodontal disease or patients using daily doses of medication, under sedatives, tranquilizers, analgesic, anticonvulsants or anti-inflammatory medication within the past 72 h were excluded. All patients who had undergone any kind of desensitizing treatment during the previous three months of their consultation were also excluded. In addition, patients who underwent two different laser-assisted protocols for two different teeth were excluded, to avoid any bias in the results.

### 2.3. Assessment and Follow-Up

Pain arising from DH was assessed before any intervention (T0), immediately after intervention (T1), one week after intervention (T2), four weeks after treatment (T3), six months after (T4) and one year after intervention (T5). In order to produce an objective assessment scale, the same procedure was used for all patients and the procedure was based on the Schiff cold air score [26]. The protocol consisted of an evaporative stimulus with air (air pressure of 4–4.5) directed from a three-in-one tip of a dental syringe, at 90° to the relevant area of the tooth, from a distance of approximately 1 cm for 1 s. The adjacent tooth was shielded using a gloved finger [26]. The patient was invited to measure the severity of their pain using a standardized visual analogue scale (VAS). In the VAS used, 0 represented no pain at all and 10 represented the greatest pain. Follow-up was carried out at T0, T1, T2, T3, T4 and T5.

### 2.4. Treatment Protocol and Laser Irradiation

#### 2.4.1. Photobiomodulation Therapy (PBM Group)

**Figure 1:**
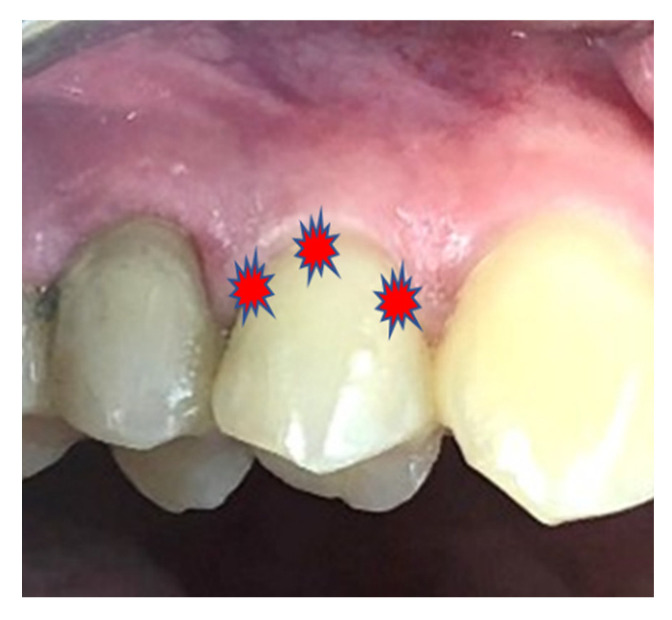
Illustration of the points of irradiation made in the PBM group.

A total of 387 teeth with DH were analyzed in the PBM group. For this group, a total of four sessions were carried out in two weeks: two sessions per week with 48 h of rest interval between each session. The light source used was a diode laser (red light) (Duo Laser, MMO, Dao Carlos, Brazil) with 660 nm wavelength. Three points of irradiation around the relevant tooth area were made on the labial side: distal, middle and mesial of the exposed dentin (three points) (Figure 1). The irradiation was always performed in contact and continuous mode with parameters of 0.1 W, 2 J, a total irradiation time of 20 s per point (60 s per session), a spot area of 0.19 cm^2^ delivered by a tip with a diameter of 0.5 cm, irradiance per point of 0.52 W/cm^2^ and energy density of 10.4 J/cm^2^.

#### 2.4.2. Nd:YAG Laser Group

**Figure 2:**
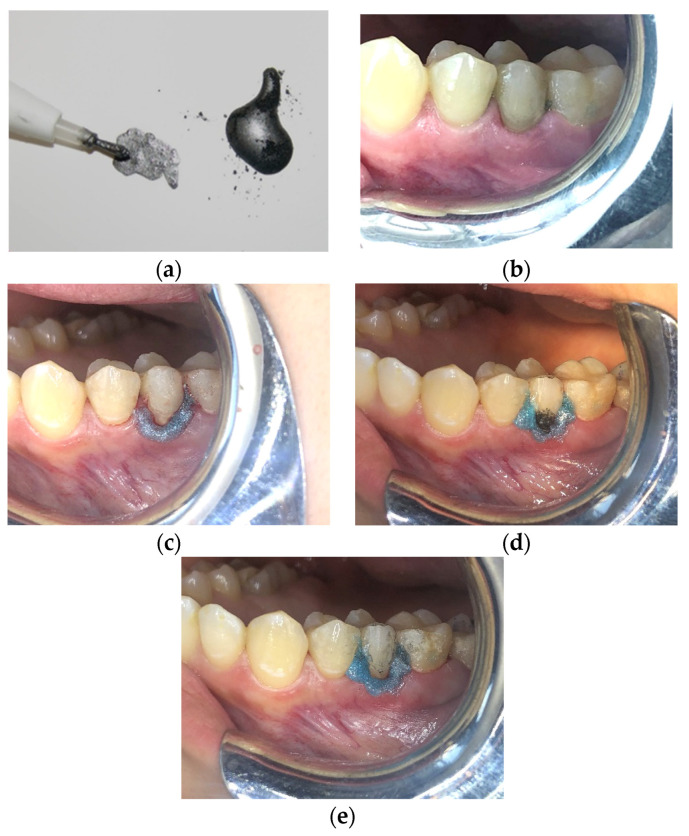
Illustration of the preparation and coating with graphite paste for the Nd:YAP and Nd:YAG groups. (**a**) Preparation of the graphite paste by mixing distilled water with graphite macrocrystalline graphite. (**b**) Pre-operative photo of the exposed dentin on the right second maxillary premolar. (**c**) Application and polymerization of the liquid rubber dam after scaling and root planing. (**d**) Application of the graphite paste only at the surface to be irradiated. (**e**) The aspect of the surface after irradiation. The absence of the graphite paste is noted.

A total of 327 teeth with DH were collected in Nd:YAG group. For this group, Nd:YAG laser with 1064 nm wavelength (Fotona Fidelis Plus II™, Ljubljana, Slovenia) were used. Before irradiation, a macrocrystalline graphite powder (Pressol graphite, Nurnberg, Germany) was mixed with distilled water until obtaining a consistency of paste. The paste obtained was used to coat the surface of the particular exposed dentin to be treated. The coating of the surface was performed using a microbrush (Figure 2a). Then, the Nd:YAG laser was used with the follow parameters: 0.5 W, frequency of 10 Hz, energy of 0.05 J, spot diameter of 300 µm, pulse duration of 120 µsec, energy density at the end of the tip of 70.77 J/cm^2^, power density of 707.71 W/cm^2^ and a pick power of 416.8 W. The irradiation speed was approximately 1 mm per second in non-contact mode and the beam was used in tangential incidence (±45°) to the surface of the relevant dentin with a distance of 1 to 2 mm between the optical fiber and the irradiated surface. The treatment was considered completed when a total vaporization of the graphite was observed (approximately 15 s). The exact same procedure was repeated for a second time and in the same session (two repeated procedures in one session) (Figure 2).

#### 2.4.3. Nd:YAP Laser Group

A total of 206 teeth with DH were collected in Nd:YAG group. For this group, Nd:YAP with 1340 nm wavelength (LOKKI, Lokki Dt., Vienne, France) was used. Graphite paste was applied exactly in the same way as for the Nd:YAG group. After applying graphite paste, Nd:YAP laser emitting at 1340 nm wavelength (LOKKI, Lokki Dt., Vienne, France) was used with the follow parameters: fiber diameter 320 µm, output power of 3 W, Energy of 300 mJ, pulse duration of 150 µsec, 10 Hz, and an energy density of 373.6 J/cm^2^ per pulse. The irradiation speed was approximately 1 mm per second in non-contact mode and the beam was used in tangential incidence (±45°) to the surface of the relevant dentin with a distance of 1 to 2 mm between the optical fiber and the irradiated surface. The treatment was considered completed when a total vaporization of the graphite was observed. The exact same procedure was repeated for a second time and in the same session (Figure 2).

### 2.5. Statistical Analysis

Statistical analyses were carried out with Prism 5^®^ software (GraphPad Software, Inc., San Diego, CA, USA). For the analysis, *p* < 0.05 was considered statistically significant. Confidence level was proposed to be 99% with *p* < 0.001, which is highly significant. Descriptive statistics, i.e., mean and standard deviation values of the VAS scores, were calculated for the different groups before treatment, just after treatment, after one week, after six months and after one year. Moreover, repeated measures ANOVA and a nonparametric test coupled with a Dunn’s test comparing all pairs of columns test (post hoc test) were used.

## 3. Results

### 3.1. Comparison between Groups

A statistically significant difference in VAS score values was obtained when different groups were compared to each other from T1 and until T3. Moreover, immediately after treatment, a statistically significant difference was obtained between all groups, with the most significant decrease in pain obtained with the Nd:YAG group (2.915 ± 0.963), followed by the PBM group (4.23 ± 0.710) and then the Nd:YAP group (6.15 ± 0.563). Moreover, from week four, the PBM group had the highest decrease in pain (0 ± 0), followed by the Nd:YAG laser; the least decrease in pain was obtained with the Nd:YAP group (4.665 ± 0.674) (Table 1).

### 3.2. Photobiomodulation Group

**Figure 3:**
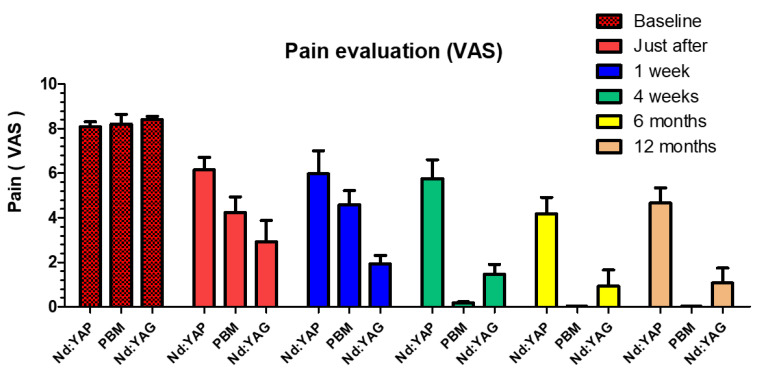
Results of the mean value and standard deviation of VAS for the three groups and at different follow-up times.

For the PBM group, the mean values of VAS were 8.197 ± 0.451 > 4.23 ± 0.710 > 4.576 ± 0.643 > 0.173 ± 0.049 > 0 and 0 at T0, T1, T2, T3, T4 and T5, respectively (Table 1). The mean VAS score values were stable, without significant difference from four weeks after treatment and until twelve months of follow-up. On the other hand, a statistically significant difference was obtained between T1 compared to all the other times of follow-up (Table 1, Figure 3).

### 3.3. Nd:YAG Group

For the Nd:YAG group, a statistically significant decrease in mean VAS score was obtained between baseline (T0) and all other times of follow-up (T1, T2, T3, T4 and T5). The decrease was stable from one week and until twelve months of follow-up (no statistical difference between values from T2 to T5 were observed). Mean VAS score values were 8.397 ± 0.150, 2.915 ± 0.963, 1.920 ± 0.39, 1.458 ± 0.4502, 0.918 ± 0.737 and 1.065 ± 0.674 for T0, T1, T2, T3, T4 and T5, respectively (Table 1, Figure 3).

### 3.4. Nd:YAP Group

For the Nd:YAP group, no statistically significant decrease in VAS score values were observed between T1 (immediately after treatment) until four weeks (T3) of follow-up and between values at six months (T4) and twelve months (T5) of follow-up. However, a statistically significant reduction of pain was obtained between baseline values (T0) and all other values of follow-ups (T1, T2, T3, T4 and T5) and also between values at T0, T1, T2, T3 and values at T4 and T5. Mean VAS scores values were: 8.072 ± 0.23, 6.15 ± 0.563, 5.97 ± 1.039, 5.758 ± 0.4502, 4.18 ± 0.737 and 4.665 ± 0.674 for T0, T1, T2, T3, T4 and T5, respectively (Table 2, Figure 3).

The results showed a statistically significant reduction of the mean values of VAS score before intervention compared to all times of follow-up, for the three groups included.

## 4. Discussion

This long-term retrospective study included 920 patients and revealed that dentinal hypersensitivity can be successfully treated with laser-assisted protocols using different wavelengths and within 12 months of stability. Interestingly, this study showed that the suggested laser-assisted protocol with photobiomodulation (PBM) therapy using a red-light diode laser or an Nd:YAG laser ensures a stable (one year of stability) outcome. Moreover, although the irradiation with Nd:YAP laser resulted in a significant decrease of the overall pain, the effectiveness was less significant compared to the results obtained with the Nd:YAG and PBM therapy. Hence, the null hypothesis was rejected.

In this study, lasers were used with two different approaches. The red 660 nm diode laser was used at low energy and in multiple sessions (PBM group) while the Nd:YAG and Nd:YAP lasers were used at relatively higher energy (0.5–3 W) and with the aim of achieving direct morphological change on the exposed dentin. Both protocols are believed to present their own advantages and to be effective in their own fashion. For instance, with the Nd:YAG and Nd:YAP laser (high-power protocol), the morphological change is obtained by the melting of the superficial surface of the dentin, which will close the open tubules [23,27]. This positive outcome obtained from the irradiation with the Nd:YAG and Nd:YAP laser can be explained by several factors related to the properties of the aforementioned lasers and the laser–tissue interaction obtained. In this context, Namour et al. [23] showed in an in vitro study that the Nd:YAP laser is able to fuse and melt the irradiated exposed dentin in one application. This was observed with scanning electron microscopy, and the protocol, similar to our procedure, consisted of coating the exposed dentinal surface with graphite paste before initiating the irradiation. The wavelength of both Nd:YAG and Nd:YAP lasers are very well absorbed by pigmented matter (dark matter) [27,28]. This fact was proven in the in vitro study of Namour et al. [23] when it was observed that with the same delivered energy, a better obliteration of the tubules can be obtained when graphite paste was used compared to the surfaces without graphite paste [23]. Smearing with graphite paste enhances the beam absorption, maintaining the energy at the level of the graphite, which will be entirely disintegrated by the energy of the laser beam. This process will generate high temperature at the exposed dentin, producing immediate dentinal fusion and resulting in partial or complete obliteration of dentinal tubules. The second reason to use graphite paste is to stop the beam of the laser at the surface of the graphite; hence, avoiding its penetration to the pulpal tissue. This is important since the dentin presents a low absorption of the Nd:YAG and Nd:YAP wavelengths; therefore, the direct irradiation of the dentin, without graphite, might generate irritation and inflammation of the pulp by the direct absorption of the beam. Furthermore, aiming to avoid any direct irradiation on the pulpal tissue by the laser beam, the irradiation was performed in tangential incidence (not perpendicular to the surface). Moreover, the melted dentin, causing the obliteration of the tubules and the stopping of the movement of the fluids, seems to resist, for 12 months, the action of wear. Additionally, previous studies using irradiation conditions similar to ours [10,23] have shown that this protocol does not produce cracks or fissures in irradiated dentin (scanning electron microscopy analysis).

Moreover, for the Nd:YAG group, although it was only a one-session intervention (with two passages), a significant reduction of the pain values from the first week and until 12 months of follow-up was produced. Additionally, our results from the PBM and Nd:YAG groups showed a progressive decrease in pain up to four weeks of follow-up. Probably, a relatively slow process occurred up to four weeks after treatments, during which eventual reduction in inflammation of the pulp was initiated after the absence of irritation by the obliteration of the dentinal tubules. Before treatment, the opened dentinal tubules were the cause of the movements of the fluids and, therefore, the cause of the local chronic inflammation of the pulpal tissue [3]. The obliteration of the dentinal tubules that was performed resulted in the elimination of the cause of the inflammation and might have activated the healing process and the reduction of the inflammation of the pulp, which is relatively slow [29,30]. This explains the decrease in pain values, even without intervention, from T1 (just after treatment) to T3 (four weeks after intervention).

Concerning the PBM group, the 660 nm diode laser was used for photobiomodulation therapy. PBM is the therapeutic use of light in order to modulate a non-thermal reaction of the living tissues [31,32,33,34,35,36]. In dentinal hypersensitivity, the PBM mechanism of action seems to be based on the biomodulation of the cellular responses, which promotes a decrease in pain levels through the depolarization of nerve fibers, on the one hand, and through the increase of tertiary dentin formation, on the other hand [37]. This will eventually lead to a significant decrease of the VAS pain values [37]. In fact, VAS values were gradually decreasing in the PBM group, which also suggests that an ongoing process was stimulated during treatment and continued to occur as time elapsed; this process is believed to be the tertiary dentin formation. Therefore, the parameters suggested in this study for PBM therapy seems to be valid for an effective modulation of pulpal inflammation. The complete attenuation of pain was stable from six months until one year of follow-up.

Another important finding from this study is that, despite using a similar procedure for the Nd:YAG and Nd:YAP lasers, the Nd:YAG laser resulted in a significantly better outcome in terms of pain reduction, suggesting that a better obliteration of the dentinal tubules was obtained with the Nd:YAG laser. The Nd:YAG laser, with its 1064 nm wavelength, is absorbed more in the pigmentation than the Nd:YAP laser. This means that when the photons from the Nd:YAG beam interact with the graphite paste (pigmentation) covering the dentinal surface, the energy of the photons will be more strongly absorbed by the pigmentation, generating more local heat, and thus producing greater dentinal fusion and more tubule obliteration. Conversely, with the Nd:YAP laser, this dentinal fusion was sufficient to cause a decrease in pain, but less than that obtained with the Nd:YAG laser, due to the lower energy absorption by the graphite paste.

The treatment of DH is widely described in the literature; however, a recent systematic review has shown that more consistent studies should be conducted to adequately observe the beneficial therapeutic effects of DH treatments. Consistent with our study, several authors have shown promising results when using lasers. Fornaini et al. [38] reported, in an in vitro study, that the 1.4 W Nd:YAP laser is safe and effective in obliterating tubules. Other laser wavelengths, not included in our retrospective study, have also been reported. El Mobadder et al. [10] used a 980 nm diode laser for the treatment of DH. They also used graphite paste and they found that a significant reduction in pain can be observed within six months of follow-up [10]. With some similarities to our procedural settings, El Mobadder et al. [10] irradiated concerned hypersensitive areas in non-contact and in tangential incidence, and they indicated that the treatment was considered accomplished when a total vaporization of the graphite was observed. Similarly, Maamary et al. [39] reported that irradiation with the Nd:YAG laser and use of graphite paste to cover the area to be treated showed significant and immediate pain relief after treatment, with no side effects [39]. Moreover, a randomized clinical trial (RCT) [12] showed that no significant difference was found between the use of 3% potassium nitrate gel and photobiomodulation therapy. In their RCT [12], PBM therapy was applied in three sessions with an 808 nm diode laser and an energy of 1J /point performed at the same points used in our study [12]. The Er,Cr:YSGG laser has been used for the treatment of DH, and several studies has reported its effectiveness [40,41,42,43,44,45]. AlHabdan et al. [40] suggested, in a systematic review, that the application of an Er,Cr:YSSG laser is effective in alleviating DH and may have limited adverse effects if adequate parameters are followed [40]. However, not all data suggest the presence of clear evidence on the effectiveness of lasers in DH treatment. A meta-analysis compared the effectiveness of different topical desensitizing agents versus lasers for the treatment of DH. The meta-analysis established that the evidence for or against the superior efficacy of lasers compared to topical desensitizing agents is weak and insufficient to draw any conclusion [45]. Our results showed a significant reduction in DH, similar to results obtained by several desensitizing agents. The difference appears in the longevity of the treatment. Nd:YAG laser and PBM showed a significant DH reduction that lasted for 12 months, while desensitizing agents (non-laser) seem to be effective for a short application period. Hypersensitivity reappears when the patient stops using the desensitizing agent [11]. In addition, Butera et al. [46] concluded in a study that the use of a toothpaste containing hydroxyapatite could be proposed as a reliable device for the domiciliary management of WSLs because of its efficacy in reducing hypersensitivity more effectively than conventional fluoride toothpaste [46].

Regardless of the pain management approach for dentin hypersensitivity, a diagnosis of the etiology, such as excessively acidic diet, improper toothbrushing, malocclusion, periodontal disease, or any associated bad habits, should be addressed before any intervention or treatment [47]. Our retrospective study shows that the described laser-assisted protocols are promising and effective for the treatment of dentin hypersensitivity. However, further studies with different wavelengths are still needed and studies with different methods, such as the use of local desensitizer, should also be conducted, and over a longer period of time. Moreover, it is important to highlight those factors such as ethnicity, gender, the status of a person and their psychological condition might affect the perception of pain, which might, in turn, affect the results of this retrospective study. For this reason, studies comparing the perception of pain arising from dentinal hypersensitivity in different ethnicities or between genders might be of interest. One shortcoming of the visual analogue scale is that patients can assess differently their level of pain and the psychological perceptive of pain can somehow affect the results.

## 5. Conclusions

Within one year of follow-up and within the limitations of this retrospective study, it was revealed that PBM therapy and irradiation with an Nd:YAG laser are effective in the management of pain arising from dentinal hypersensitivity. The Nd:YAP laser reduced pain but did not effectively manage pain arising from dentinal hypersensitivity. PBM therapy was the only therapeutic procedure that led to a complete absence of pain at six to twelve months after treatment. Moreover, the least pain reduction at twelve months of follow-up was obtained with the Nd:YAP laser.

## Figures and Tables

**Table 1:** Clinical features of the included participants.

Total Participants	Total Teeth	Gender		Mean Age (Years)	Ethnicity		
811	920	Male	Female	47 (Min: 38; Max: 65)	South American	Caucasian	Middle Eastern
		233	578		294	422	95

**Table 2:** Mean and standard deviation of VAS scores for all groups at different times of follow-up. The mean of VAS values at baseline represents the average of total VAS values in all groups at baseline.

		Baseline (T0)	Immediately after (T1)	1 Week (T2)	4 Weeks (T3)	6 Months (T4)	12 Months (T5)
PBM (*n* = 387)	mean	8.197 ^A^	4.23 ^B^	4.576 ^B^	0.173 ^C^	0 ^C^	0 ^C^
	Std.	0.451	0.710	0.643	0.049	0	0
Nd:YAG (*n* = 327)	mean	8.397 ^A^	2.915 ^D^	1.920 ^E^	1.458 ^E^	0.918 ^E^	1.065 ^E^
	Std.	0.150	0.963	0.39	0.4502	0.737	0.674
Nd:YAP (*n* = 206)	mean	8.072 ^A^	6.15 ^F^	5.970 ^F^	5.758 ^F^	4.18 ^B^	4.665 ^B^
	Std.	0.230	0.563	1.039	0.4502	0.737	0.674

Different superscript letters indicate statistically significant difference (^A, B, C, …^); similar superscript letters indicate the absence of a statistically significant difference (^A; A or B; B…^). mean = mean value; std. = standard deviation.

## Data Availability

Data available upon reasonable request.
